# “Out, out, brief candle! Life’s but a walking shadow”: *5-HTTLPR* Is Associated With Current Suicidal Ideation but Not With Previous Suicide Attempts and Interacts With Recent Relationship Problems

**DOI:** 10.3389/fpsyt.2020.00567

**Published:** 2020-06-25

**Authors:** Janos Bokor, Sandor Krause, Dora Torok, Nora Eszlari, Sara Sutori, Zsofia Gal, Peter Petschner, Ian M. Anderson, Bill Deakin, Gyorgy Bagdy, Gabriella Juhasz, Xenia Gonda

**Affiliations:** ^1^ Department of Forensic and Insurance Medicine, Semmelweis University, Budapest, Hungary; ^2^ Nyírő Gyula National Institute of Psychiatry and Addictions, Budapest, Hungary; ^3^ Department of Pharmacodynamics, Faculty of Pharmacy, Semmelweis University, Budapest, Hungary; ^4^ NAP-2-SE New Antidepressant Target Research Group, Hungarian Brain Research Program, Semmelweis University, Budapest, Hungary; ^5^ Faculty of Humanity and Social Sciences, Institute of Psychology, Pazmany Peter Catholic University, Budapest, Hungary; ^6^ MTA-SE Neuropsychopharmacology and Neurochemistry Research Group, Hungarian Academy of Sciences, Semmelweis University, Budapest, Hungary; ^7^ Neuroscience and Psychiatry Unit, Division of Neuroscience and Experimental Psychology, School of Biological Sciences, Faculty of Biological, Medical and Human Sciences, The University of Manchester and Manchester Academic Health Sciences Centre, Manchester, United Kingdom; ^8^ SE-NAP-2 Genetic Brain Imaging Migraine Research Group, Semmelweis University, Budapest, Hungary; ^9^ Department of Psychiatry and Psychotherapy, Semmelweis University, Budapest, Hungary

**Keywords:** suicide attempts, suicidal ideation, childhood adversities, recent life events, *5-HTTLPR*

## Abstract

**Background:**

Suicide is an unresolved psychiatric and public health emergency, claiming 800,000 lives each year, however, its neurobiological etiology is still not understood. In spite of original reports concerning the involvement of *5-HTTLPR* in interaction with recent stress in the appearance of suicidal ideation and attempts, replication studies have yielded contradictory results. In our study, we analyzed the association between *5-HTTLPR* and lifetime suicide attempts, current suicidal ideation, hopelessness and thoughts of death as main effects, and in interaction with childhood adversities, recent stress, and different types of recent life events in a general population sample.

**Methods:**

Two thousand and three hundred fifty-eight unrelated European volunteers were genotyped for *5-HTTLPR*, provided phenotypic data on previous suicide attempts, and current suicidal ideation, hopelessness and thoughts about death, and information on childhood adversities and recent life events. Logistic and linear regression models were run with age, gender, and population as covariates to test for the effect of *5-HTTLPR* as a main effect and in interaction with childhood adversities and recent life events on previous suicide attempts and current suicidal ideation. Benjamini-Hochberg FDR Q values were calculated to correct for multiple testing.

**Results:**

*5-HTTLPR* had no significant effect on lifetime suicide attempts either as a main effect on in interaction with childhood adversities. *5-HTTLPR* had a significant main effect on current suicidal ideation in the dominant model (Q=0.0344). *5-HTTLPR* did not interact with childhood adversities or total number of recent life events on any phenotypes related to current suicidal risk, however, a significant interaction effect between *5-HTTLPR* and current relationship problems emerged in the case of current suicidal ideation in the dominant model (Q=0.0218) and in the case of thoughts about death and dying in the dominant (Q=0.0094) and additive models (Q=0.0281).

**Conclusion:**

While *5-HTTLPR* did not influence previous suicide attempts or interacted with childhood adversities, it did influence current suicidal ideation with, in addition, an interaction with recent relationship problems supporting the involvement of *5-HTTLPR* in suicide. Our findings that *5-HTTLPR* impacts only certain types of suicide risk-related behaviors and that it interacts with only distinct types of recent stressors provides a possible explanation for previous conflicting findings.

## Introduction

Suicide remains an unresolved psychiatric and public health emergency, claiming 800,000 lives each year, contributing to one death happening every 40 seconds (1), and it is the second leading cause of death in adolescents and young adults in the 15–29 age group (1). In spite of declining trends worldwide, in young people in the United States suicide shows an alarmingly increasing rate (2) which, in spite of decades of research aimed at uncovering its biopsychosocial aspects and determinants highlights that we are still far from understanding, and even further from effectively predicting and preventing suicidal behaviors.

Suicidal behavior occurs along a spectrum from thinking about suicide or suicidal ideation, planning, suicidal attempts, aborted suicides to completed suicide (3), all of which cause significant suffering and burden. While 90% of completed suicides occur in psychiatric patients (4), the majority of whom suffer from affective disorders where it may be expected and thus targeted, still quite a few nonpsychiatric subjects resort to suicidal behavior. While suicidal behaviors, especially attempts and completed suicides, cannot be predicted, one possible marker heralding approaching suicide may be the appearance of suicidal thoughts, which are often the threshold of the downward spiral leading to more lethal suicidal manifestations. While the frequency of suicide attempts is up to 20 times that of completed suicides with a lifetime prevalence of 2.7%, suicidal ideation has a 9.2% lifetime prevalence, with 29% of ideators (and 56% of ideators with a plan) proceeding to making a subsequent suicidal attempt (5). Thus, while there may be some heterogeneity not only in the manifestation but also in the neurobiology of suicidal behavior along the suicide spectrum (6, 7), due to completed suicide being a relatively rare event in the community other manifestations along the spectrum are used in studies to extrapolate and estimate suicide risk (3).

Also, due to the significant suffering and burden associated with suicidal behavior coupled with the impossibility to reliably and effectively predict suicide as declared by the American Psychiatric Association and the Institute of Medicine (8), there has been a surge to identify predictors, such as suicidal ideation as well as biomarkers including genetic variants that predict suicidality.

Family, adoption, and twin studies clearly show a substantial role of genetic factors in suicidal behavior, contributing to a heritability estimated between 30% and 55% in different studies (9, 10), with an average of 43%, which is in general comparable to the heritability of psychiatric illnesses (11, 12) but is at least in part independent of the genetic transmission of affective disorders (13). There is a ten-fold risk of suicidal behavior in relatives of suicide completers (14) and a 175-time relative risk for suicidal behaviors including attempts and completion in monozygotic twin pairs compared to dizygotic ones (15).

Due to initial findings of an association between low 5-HIAA levels in the cerebrospinal fluid (16, 17) of suicidal depressive patients and subsequent reports of lower serotonin transporter expression in various relevant regions of the suicidal brain (18, 19), the serotonergic system has been early implicated in suicidal behavior and subsequently genes in the serotonergic system have become prime candidates in the search for the genetic underpinnings of suicide. Already in the early era of psychiatric genetics, attention was focused on *5-HTTLPR*, a 44-base pair insertion-deletion polymorphisms (rs4795541), located upstream from the transcription start site in the promoter region and playing a role in the regulation of the serotonin transporter gene *SLC6A4*, located on chromosome 17 (17q11.2) and encoding a presynaptic transmembrane protein responsible for the reuptake of serotonin. The 14-repeat short variant (s allele) of *5-HTTLPR*, contributing to reduced expression compared to the 16-repeat long variant (l allele) (20, 21) has been found to be associated with several phenomena related to the appearance of suicidal behavior including depression (22), affective temperaments (23), aggression (24–26), and impulsivity (27–29), especially in the context of environmental stress. *5-HTTLPR* has also been implicated in the background of personality traits involved in increased risk of suicidal ideation, including for example alexithymia (30, 31), which could prove useful in prediction, screening and management of suicide risk (32–34). While there has been a large number of subsequent studies focusing on the role of *5-HTTLPR* in suicide and related risk factors, there is a lack of agreement due to the contradictory findings of these studies.

In addition to the substantial heritable contribution to suicidal behavior, and in line with the commonly accepted diathesis-stress model of suicide (35), in addition to genetic factors there is also an equally substantial role for environmental components including both predisposing distal and precipitating proximal factors, which emphasises the role of both early and current adverse experiences (7), and plays a key role in modulating the genetic predisposition and triggering of suicide (36). In spite of this, suicidal behaviors have been less frequently studied in gene x environment models, although successful prevention of suicide would require understanding of how risk of suicide emerges in the presence of biological predispositions triggered by external influences. In spite of Caspi and colleagues’ initial paper (22) on the interaction of serotonin transporter gene with life events involved not only in depression but also suicide, only a few studies approached the role of *5-HTTLPR* in suicidal behavior with gene x environment interaction models.

Thus, in our present study, we analyzed the impact of *5-HTTLPR* on previous suicide attempts, current suicidal ideation, and other suicide risk markers such as hopelessness and death-related thoughts in interaction with early childhood adverse experiences and recent negative life events in a large general European population.

## Materials and Methods

### Participants

This investigation is part of the NewMood project (New Molecules in Mood Disorders, Sixth Framework Program of the EU, LSHM-CT-2004-503474). In total, 2,588 non-related, European white ethnic origin volunteer participants (1,808 female and 780 male) aged between 18 and 60 from Greater Manchester and Budapest recruited between 2005–2008 through general practices or *via* the internet (www.newmood.co.uk) were sent a questionnaire pack and a genetic sampling kit. Inclusion criteria included voluntary participation, signing of informed consent, providing genetic material and returning the questionnaire pack. Inclusion was independent of any positive psychiatric anamnesis. Subjects whose DNA sample was not successfully genotyped as well as subjects with missing questionnaire data were excluded from statistical tests. The present analysis was carried out in 2,358 subjects who provided eligible phenotypic data and could be genotyped for *5-HTTLPR*. More details about the population sample can be found in our previously published reports (37–39). The study has been conducted in accordance with the Declaration of Helsinki and has been approved by the local ethics committees. All subjects gave written informed consent prior to participation in the study.

### Phenotypes

Previous suicidal attempts (SUIC) were recorded through self-report. Current markers of suicidal ideation were measured *via* relevant items of the Brief Symptom Inventory (40), including item 3, “Thoughts of ending your life” to indicate suicidal ideation (BSI03), as well as item 18, “Feeling hopeless about the future”, to indicate hopelessness (BSI18), a well-established independent predictor of suicide risk, and item 21, “Thoughts about death and dying” to indicate death-related thoughts (BSI21) during the previous week using a scale of 0–4. Childhood adversities (CHA) were measured using a short form of the Childhood Trauma Questionnaire (CTQ) (41) and covered emotional and physical abuse and emotional and physical neglect. Recent negative life events (RLE) occurring over the previous 12 months were measured by the List of Threatening Experiences (42) which sums life events related to four validated subscales (43) previously used in gene-by-environment interaction models (GxE) (44, 45). The four subscales include financial difficulties (RLE-financial), personal problems (RLE-personal), intimate relationship problems (RLE-relationship), and social network disturbances (RLE-social). Intercorrelations between the subscales have also been previously reported and were found to be either nonsignificant (between RLE-social and RLE-financial; and RLE-social and RLE-relationship), or significant but negligible weak (44).

### Genetic Data

The extraction of the DNA from buccal mucosa cells was based on previously applied protocol (46). For the *5-HTTLPR* genotype characterization we used Illumina CoreExom PsychChip as described elsewhere (38). The quality control and imputation steps are based on our previous paper (38). All laboratory work was performed under the ISO 9001:2000 quality management requirements and was blinded with regard to phenotype.

### Statistical Analysis

IBM SPSS Statistics 25 was used to calculate descriptive statistics and to run univariate general linear models solely for visualization purposes. Plink v1.90 (https://www.cog-genomics.org/plink2) was used to calculate Hardy-Weinberg equilibrium and minor allele frequency (MAF), and to build dominant, recessive and additive linear and logistic regression models with age, gender, and population as covariates in all models. Analyses were supported by scripts individually written in R 3.0.2 (R Core Team, 2013). In case of each outcome variable including lifetime SUIC, current suicidal ideation (BSI03-Thoughts of ending your life), current hopelessness (BSI18-Feeling hopeless about the future), and current thoughts of death (BSI21-Thoughts about death and dying), first the main effect of *5-HTTLPR* was tested, followed by interaction analyses. Interaction analyses included interaction with CHA in case of SUIC, and interaction with CHA, RLE, and subtypes of RLE including recent relationship problems (RLE-relationship), recent financial problems (RLE-financial), recent personal problems (RLE-personal), and recent social network problems (RLE-social) in case of BSI03, BSI18, and BSI21. All analyses were run according to additive, dominant, and recessive models. Nominal significance threshold was p < 0.05. To correct for multiple comparisons in analyses for each of the above outcome variables, Benjamini-Hochberg false discovery rate (FDR) Q-values (without robust method) were calculated. Results with a Q-value ≤ 0.05 were considered as significant. Raw data comprising the basis of the presented analyses are available at https://doi.org/10.6084/m9.figshare.12214748.v1 (47). Only in order to facilitate visualization in the general linear models, RLE scores were divided into three categories as described previously (44, 45) as 0 event, 1 event, 2 or more events.

## Results

### Descriptive Data

The descriptive data of our study population are shown in **Table 1**.

**Table 1:** Description of the study population.

Demographics				
**Gender**	Males: 723 (30.66%) Females: 1635 (69.33%)			
**Age**	Minimum	Maximum	Mean	SEM
	18	60	32.80	0.2190
**Phenotypes**				
**Previous suicide attempts**	No: 2074 (88%)			
	Yes: 284 (12%)			
**Current suicidal risk factors**				
*L1BSI03 (thoughts of ending your life)*	0	4	0.32	0.0199
*L1BSI18 (feeling hopeless about the future)*	0	4	0.90	0.0295
*L1BSI21 (thoughts of about death and dying)*	0	4	0.62	0.0262
**Environmental stressors**				
**Childhood adversities**	0	16	3.29	0.0694
**Recent Life Events**	0	8	1.22	0.0266
*RLE-relationship*	0	2	0.14	0.0080
*RLE-financial*	0	3	0.21	0.0106
*RLE-personal problems*	0	3	0.36	0.0128
*RLE-social*	0	3	0.41	0.0133
**Genotypes**				
Ss	438 (18.58%)			
Sl	1138 (48.26%)			
Ll	782 (33.16%)			
**MAF (minor allele frequency)**	0.43 (s)			

BSI, Brief Symptom Inventory; RLE-relationship, intimate relationship problems; RLE-financial, financial difficulties; RLE-illness, Illness/injury; RLE-social, social network disturbances.

*5-HTTLPR* was in Hardy-Weinberg equilibrium in our total sample (p=0.8942). Minor allele frequency was 0.4271 for the short (s) allele.

Gene-environment correlations, as calculated in linear regression models for RLE and its subscales and for CHA, are shown in **Table 2**. *5-HTTLPR* genotype had no significant effect on CHA, RLE, or on individual recent life event subtypes (**Table 2**).

**Table 2:** Main effect of *5-HTTLPR* on the occurrence of environmental stressors.

	ADD		DOM		REC	
	p	FDR Q	P	FDR Q	p	FDR Q
CHA	0.3402	0.4862	0.5502	0.5502	0.2885	0.5709
RLE whole	0.1044	0.4534	**0.0343**	0.2058	0.3660	0.5709
RLE-relationship	0.7481	0.7481	0.5257	0.5502	0.5371	0.6445
RLE- financial	0.2267	0.4534	0.0868	0.2160	0.6905	0.6905
RLE-personal problems	0.4052	0.4862	0.2098	0.3147	0.3806	0.5709
RLE-social	0.2076	0.4534	0.1080	0.2160	0.2128	0.5709

CHA, childhood adversities; RLE, recent life events; p values for linear regression models are shown with FDR Q correction for multiple testing. **Bold** type denotes nominally significant (p < 0.05) values prior to correction.

### Investigation of the Main Effect of *5-HTTLPR* on Lifetime Suicide Attempts, Current Suicidal Ideation, and Suicide Risk Indicators

No main effect of *5-HTTLPR* was detectable on previous SUIC in additive (Q=0.7134), dominant (Q=0.7134), or recessive models (Q=0.7134) (**Table 3**). *5-HTTLPR* had a significant main effect on BSI03 Thoughts of ending your life in the dominant (Q=0.0344) model with presence of s allele associated with higher scores, but not in additive (Q=0.0669) or recessive (Q=0.8532) models (**Table 4**). No main effect of *5-HTTLPR* on other current markers of suicidal risk including BSI18 Feeling hopeless about the future (Q=0.4320, Q=0.4320, Q=0.4792 for additive, dominant, and recessive models, respectively) (**Table 5**) or BSI21 Thoughts about death and dying (Q=0.1872, Q=0.4083, Q=0.1872 for additive, dominant, and recessive models, respectively) emerged in any of the models (**Table 6**).

**Table 3:** Main effect of *5-HTTLPR* and interaction with childhood adversities (CHA) on previous suicide attempt (SUIC).

*5-HTTLPR*	ADD				DOM				REC			
**	OR	SE	P	FDR Q	OR	SE	P	FDR Q	OR	SE	P	FDR Q
Main effect	1.049	0.0926	0.6058	0.7134	1.068	0.1405	0.6392	0.7134	1.062	0.1649	0.7134	0.7134
Interaction with CHA	1.178	0.1086	0.1312	0.4812	1.261	0.165	0.1604	0.4812	1.236	0.1964	0.2802	0.5604

CHA, childhood adversities; ADD, additive model; DOM, dominant model; REC, recessive model.

**Table 4:** Main effect of *5-HTTLPR* and interaction with childhood adversities (CHA) on current suicidal ideation (L1BSI03 Thoughts of ending your life).

*5-HTTLPR*	ADD				DOM				REC			
	Beta	SE	P	FDR Q	Beta	SE	P	FDR Q	Beta	SE	P	FDR Q
Main effect	0.07152	0.03127	0.02229	0.0669	0.1293	0.0468	**0.0057**	***0.0344***	0.0451	0.0568	0.4266	0.8532
Interaction with CHA	0.0025	0.0386	0.9457	0.9580	0.0030	0.0573	0.9580	0.9580	0.0067	0.0699	0.9234	0.9580

CHA, Childhood adversities; ADD, additive model; DOM, dominant model; REC, recessive model.

**Bold** type denotes nominally significant (p < 0.05) values prior to correction; **bold italics** indicate significant p values surviving correction for multiple testing (p < 0.05, FDR Q < 0.05).

**Table 5:** Main effect and interactions of *5-HTTLPR* with childhood adversities (CHA) on current hopelessness (L1BSI18 Feeling hopeless about the future).

*5-HTTLPR*	ADD				DOM				REC			
	Beta	SE	P	FDR Q	Beta	SE	P	FDR Q	Beta	SE	P	FDR Q
Main effect	0.0608	0.0374	0.1041	0.4320	0.0818	0.0560	0.1440	0.4320	0.0797	0.0678	0.2396	0.4792
Interaction with CHA	-0.0134	0.0455	0.7680	0.9216	-0.0248	0.0678	0.7148	0.9216	-0.0049	0.0822	0.9524	0.9524

CHA, Childhood adversities; ADD, additive model; DOM, dominant model; REC, recessive model.

**Table 6:** Main effect and interactions of *5-HTTLPR* with childhood adversities (CHA) on current thoughts of death (L1BSI21 Thoughts about death and dying).

*5-HTTLPR*	ADD				DOM				REC			
	Beta	SE	P	FDR Q	Beta	SE	P	FDR Q	Beta	SE	P	FDR Q
Main effect	0.0489	0.0313	0.1188	0.1872	0.0388	0.0469	0.4083	0.4083	0.1035	0.0567	0.0681	0.1872
Interaction with CHA	0.0633	0.0382	0.0978	0.1872	0.0694	0.0569	0.2229	0.2675	0.1062	0.0692	0.1248	0.1872

CHA, Childhood adversities; ADD, additive model; DOM, dominant model; REC, recessive model.

### Interaction Effect of *5-HTTLPR* and Childhood Adversities (CHA) on Lifetime Suicide Attempts, Current Suicidal Ideation, and Current Predictors of Suicide Risk

*5-HTTLPR* had no significant interaction effect with childhood adversities on previous SUIC in any of the models (Q=0.7134, Q=0.7134, Q=0.7134, in additive, dominant, and recessive models, respectively) (**Table 3**). Similarly, there was no significant *5-HTTLPR* x CHA interaction effect detectable on current suicidal ideation (BSI03 Thoughts of ending your life) (Q=0.9580, Q=0.9580, Q=0.9580 for additive, dominant, and recessive models, respectively) (**Table 4**); on current hopelessness (BSI18 Feeling hopeless about the future) (Q=0.9216, Q=0.9216, Q=0.9524 for additive, dominant, and recessive models, respectively) (**Table 5**); or on current thoughts of death (BSI21 Thoughts about death and dying) (Q=0.1872, Q=0.2675, Q=0.1872, for additive, dominant, and recessive models, respectively) (**Table 6**).

### Interaction Effect of *5-HTTLPR* and Total Number of Recent Life Events (RLE) on Current Suicidal Ideation and Current Predictors of Suicide Risk

No interaction effect was observed with total number of recent life events in case of current suicidal ideation (BSI03 Thought of ending your life) (Q=0.7455, Q=0.6052, Q=0.3540, for additive, dominant, and recessive models, respectively) (**Table 7**), current hopelessness (BSI18) (Q=0.3992, Q=0.2160, Q=0.7817, for additive, dominant, and recessive models, respectively) (**Table 8**), or current thoughts about death or dying (BSI21) (Q=0.2690, Q=0.6124, Q=0.3981, for additive, dominant, and recessive models, respectively) (**Table 9**).

**Table 7:** Main effect and interactions of *5-HTTLPR* with recent life events (RLE) and RLE subtypes on current suicidal ideation (L1BSI03 Thoughts of ending your life).

*5-HTTLPR*	ADD				DOM				REC			
	β	SE	P	FDR Q	β	SE	P	FDR Q	β	SE	P	FDR Q
Main effect	0.0715	0.0313	0.0223	0.1337	0.1293	0.0468	**0.0057**	***0.0218***	0.0451	0.0568	0.4266	0.6399
Interaction with RLE	-0.0136	0.0419	0.7455	0.7455	0.0409	0.0613	0.5043	0.6052	-0.1242	0.0794	0.118	0.3540
Interaction with RLE-relationship	0.0483	0.0281	*0.0862*	0.2160	0.1212	0.0451	**0.0073**	***0.0218***	0.0071	0.0518	0.8907	0.9128
Interaction with RLE-financial	-0.0415	0.0390	0.2875	0.4312	0.0032	0.0594	0.9577	0.9577	-0.1440	0.0714	**0.0439**	0.2631
Interaction with RLE-personal	0.0427	0.0891	0.6322	0.7455	0.0923	0.1341	0.4914	0.6052	0.0180	0.1638	0.9128	0.9128
Interaction with RLE-social	0.1829	0.1132	0.1063	0.2126	0.2612	0.1683	0.1207	0.2414	0.2270	0.2122	0.2848	0.5696

RLE-relationship, intimate relationship problems; RLE-financial, financial difficulties; RLE-personal, personal problems; RLE-social, social network disturbances; ADD, additive model; DOM, dominant model; REC, recessive model. **Bold** type denotes nominally significant (p < 0.05) values prior to correction; **bold italics** indicate significant p values surviving correction for multiple testing (p < 0.05, FDR Q < 0.05).

**Table 8:** Main effect and interactions of *5-HTTLPR* with recent life events (RLE) and RLE subtypes on current hopelessness (L1BSI18 Feeling hopeless about the future).

*5-HTTLPR*	ADD				DOM				REC			
	β	SE	P	FDR Q	β	SE	P	FDR Q	β	SE	P	FDR Q
Main effect	0.0608	0.0374	0.1041	0.3992	0.0818	0.056	0.144	0.2160	0.0797	0.0678	0.2396	0.7188
Interaction with RLE	0.0634	0.0494	0.1996	0.3992	0.1084	0.0726	0.1357	0.2161	0.0467	0.0928	0.6147	0.7817
Interaction with RLE-relationship	0.04728	0.0336	0.1592	0.3992	0.0962	0.0525	0.0671	0.2162	0.0278	0.0616	0.6514	0.7817
Interaction with RLE-financial	-0.0174	0.0423	0.6814	0.6814	-0.0455	0.0630	0.4702	0.5642	0.0104	0.0774	0.8934	0.8934
Interaction with RLE-personal	-0.0482	0.0662	0.4668	0.5602	-0.1452	0.0949	0.126	0.2164	0.0712	0.1181	0.5467	0.7817
Interaction with RLE-social	0.0615	0.0729	0.3988	0.5602	0.0216	0.1134	0.8487	0.8487	0.1719	0.1318	0.1922	0.7188

RLE-relationship, intimate relationship problems; RLE-financial, financial difficulties; RLE-personal, personal problems; RLE-social, social network disturbances; ADD, additive model; DOM, dominant model; REC, recessive model.

**Table 9:** Main effect and interactions of *5-HTTLPR* with recent life events (RLE) and RLE subtypes on current thoughts of death (L1BSI21 Thoughts about death and dying).

*5-HTTLPR*	ADD				DOM				REC			
	β	SE	P	FDR Q	β	SE	P	FDR Q	β	SE	P	FDR Q
Main effect	0.0489	0.0313	0.1188	0.2690	0.0388	0.0469	0.4083	0.6124	0.1035	0.057	0.0681	0.3981
Interaction with RLE	0.0625	0.0417	0.1345	0.2690	0.1075	0.0613	0.0795	0.6124	0.0494	0.0786	0.5297	0.3981
Interaction with RLE-relationship	0.0780	0.02755	**0.0047**	***0.0281***	0.1364	0.0431	**0.0016**	***0.0094***	0.0761	0.0506	0.1327	0.3981
Interaction with RLE-financial	0.01612	0.0352	0.6469	0.7539	0.0243	0.0524	0.6436	0.6436	0.0183	0.0644	0.7762	0.9202
Interaction with RLE-personal	0.0173	0.0550	0.7539	0.7539	-0.0428	0.0788	0.5875	0.6436	0.1200	0.0982	0.2219	0.4438
Interaction with RLE-social	0.0532	0.0604	0.3786	0.5679	0.1365	0.0938	0.1460	0.2920	-0.0110	0.1093	0.9202	0.9202

RLE-relationship, intimate relationship problems; RLE-financial, financial difficulties; RLE-personal, personal problems; RLE-social, social network disturbances; ADD, additive model; DOM, dominant model; REC, recessive model. **Bold** type denotes nominally significant (p < 0.05) values prior to correction; **bold italics** indicate significant p values surviving correction for multiple testing (p < 0.05, FDR Q < 0.05).

### Interaction Effect of *5-HTTLPR* and Different Subtypes of Current Life Events on Current Suicidal Ideation and Other Current Predictors of Suicide Risk

**Figure 1:**
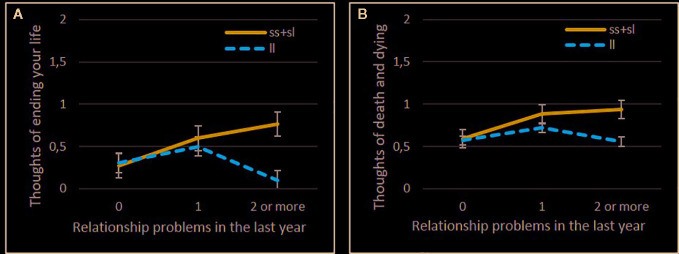
Significant effect of *5-HTTLPR* genotype in interaction with recent life events related to relationship problems in the past year (RLEC-relationship) on current suicidal ideation and thoughts about death and dying in the dominant model (ss+sl vs ll genotype). Recent life events are categorized as 0 (no recent life events reported), 1 (1 life event reported), and 2 (2 or more life events reported in the last year). Mean ± SEM is displayed. Linear regression indicated a significant interaction between *5-HTTLPR* genotype and recent life events related to relationship problems (RLE-relationship) on current suicidal ideation (BSI03 Thoughts of ending your life) (Q=0.0218) **(A)** and on current thoughts of death (BSI21 Thoughts about death or dying) (Q=0.0094) **(B)** in the dominant model. Presence of the s allele is associated with increasing severity of suicidal ideation and thoughts about death with increasing number of relationship problem experiences in the previous year.

Interaction analyses for distinct recent life event subtypes showed a strong significant effect on current suicidal ideation (BSI03 Thoughts of ending your life) in case of recent intimate relationship problems (RLE-relationship) in the dominant (Q=0.0218) (**Figure 1**), but no effect in the additive (Q=0.2160 or recessive models (Q=0.9128). No significant interaction effect for *5-HTTLPR* with other recent life event subtypes, such as recent financial hardships (RLE-financial) (Q=0.4312, Q=0.9577, Q=0.2631 for additive, dominant, and recessive models respectively); recent personal problems (RLE-personal) (Q=0.7455, Q=0.6052, Q=0.9128 for additive, dominant, and recessive models respectively); or recent social network problems (RLE-social) (Q=0.2126, Q=0.2414, Q=0.5696 for additive, dominant, and recessive models respectively) emerged in any models (**Table 7**).

In the case of current hopelessness (BSI18 Feeling hopeless about the future), no significant interaction effect emerged with any types of recent life events in any models such as RLE-relationship (Q=0.39912, Q=0.2162, Q=0.7817 for additive, dominant, and recessive models respectively); RLE-financial (Q=0.6814, Q=0.5462, Q=0.8934 for additive, dominant, and recessive models respectively); RLE-personal problems (Q=0.5602, Q=0.2164, Q=0.7817 for additive, dominant, and recessive models respectively); or RLE-social (Q=0.5602, Q=0.8487, Q=0.7188 for additive, dominant, and recessive models respectively) (**Table 8**).

In the case of thoughts about death and dying (BSI21) a strong significant effect between *5-HTTLPR* and recent relationship problems (RLE-relationship) was found in the additive (Q=0.0281) and dominant (Q=0.0094) (**Figure 1**) but not in recessive models (Q=0.3981). In case of the other three recent life event subtypes, that is recent financial hardships (RLE-financial) (Q=0.7539, Q=0.6436, Q=0.9202 for additive, dominant, and recessive models respectively); recent personal problems (RLE-Personal) (Q=0.7539, Q=0.6436, Q=0.4438 for additive, dominant, and recessive models respectively); or recent social network problems (RLE-social) (Q=0.5679, Q=0.2920, Q=0.9202 for additive, dominant, and recessive models respectively) no significant interaction effect emerged in any models (**Table 9**).

**Figure 2:**
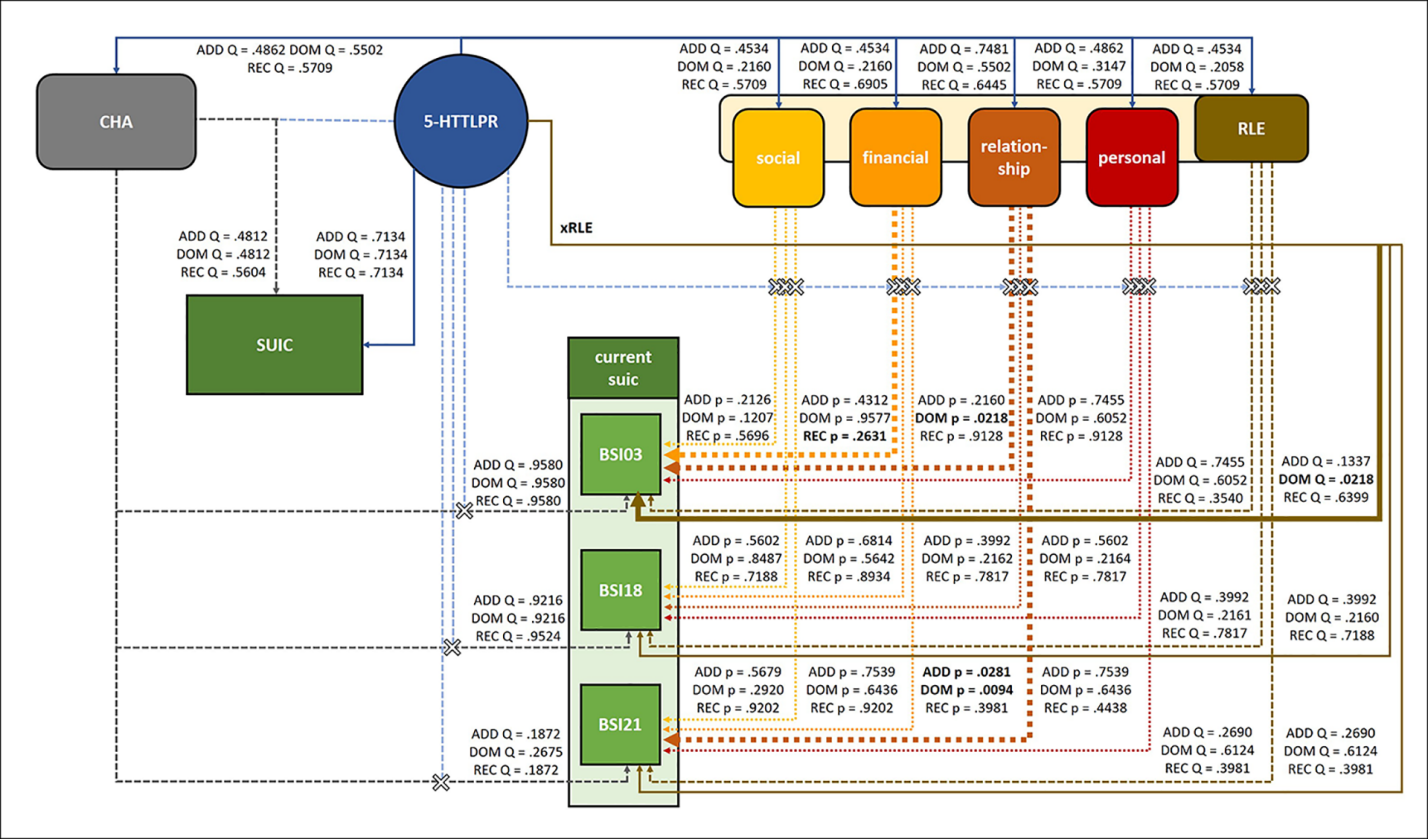
Main effects and interactions of *5-HTTLPR* with childhood adversities (CHA), recent life events (RLE) and subtypes of recent life events on lifetime suicide attempts (SUIC), current suicidal ideation (BSI03 Thoughts of ending your life), current hopelessness (BSI18 Feeling hopeless about the future), and current thoughts of death (BSI21 Thoughts about death and dying). Of the main effect and interaction effects investigated in the present study, *5-HTTLPR* had a signifcant main effect on current suicidal ideation (BSI03 Thoughts of ending your life) in the dominant model, and significantly interacted with recent relationship problems on current suicidal ideation (BSI03 Thoughts of ending your life) in the dominant and on current thoughts about death (BSI21 Thoughts about death or dying) in the dominant and additive models. In all cases, presence of the short allele was associated with higher scores. Solid lines indicate main effects, dashed lines with “X” indicate interaction effects; significant effects are indicated by bold lines and significant Q values are shown in bold type. CHA, childhood adversities; SUIC, lifetime suicide attempts; RLE, recent life events; social, recent social network stressors; financial, recent financial hardships; relationship, intimate relationship problems; personal, recent personal problems; current suic, current suicidal risk markers; BSI03, Thoughts of ending your life; BSI18, Feeling hopeless about the future; BSI21, Thoughts about death and dying.

The investigated effects including the significant results are shown in **Figure 2**.

## Discussion

Our study focused on the effect of *5-HTTLPR* on lifetime suicide attempts and current suicidal ideation and other predictors of current suicidal risk including hopelessness and thoughts of death, in interaction with childhood adverse experiences and distinct types of recent negative life events in a large general population sample. We found that *5-HTTLPR* genotype did not influence lifetime suicide attempts, but had a significant effect on current suicidal ideation, with no effect on current hopelessness or thoughts of death. In our present study, childhood adversities did not interact with *5-HTTLPR* either on lifetime suicide attempts, current suicidal ideation, or any current predictors of suicide risk. Recent life events in total also did not interact with *5-HTTLPR* on current suicidal ideation or other predictors of current suicide risk. However, when subtypes of recent stress were considered separately, *5-HTTLPR* showed a strong interaction with intimate relationship problems but not with other recent stressors on current suicidal ideation and on current thoughts about death or dying. In all cases, presence of the s allele (ss and sl genotypes) was associated with increased scores. No other types of recent life events, such us financial hardships, personal problems, or social network difficulties interacted with *5-HTTLPR* on current suicidal ideation or other predictors. Furthermore, no significant effect of *5-HTTLPR* or an interaction with early childhood adversities or recent negative life events emerged in case of hopelessness. Thus, our results suggest that *5-HTTLPR* is involved in current suicidal ideation directly and in interaction with current relationship problems.

### Presence of the s Allele of *5-HTTLPR* Is Not Associated With Increased Risk of Lifetime Suicide Attempts, but Is Associated With Current Suicidal Ideation

*5-HTTLPR* emerged as a potential genetic marker associated with suicidal behavior in part because of its association with several potentially related neural, clinical, and personality characteristics including hippocampal volume (48), amygdala reactivity (49, 50), personality traits such as neuroticism (24), anxiety (21), affective temperaments (23), aggression (25), and a range of psychiatric disorders beyond depression including anxiety disorders, substance use disorders, ADHD, autism, eating disorders, and psychosomatic disorders suggesting that *5-HTTLPR* mediates a nonspecific vulnerability for mental and behavioral disturbances, and specifically plays a role in modulating the effects of environmental influences in the development of mental problems (51). Following a number of controversial results from individual studies, meta-analyses of *5-HTTLPR* in suicidal behavior have been carried out yielding equally contradicting results, with no results reported in some (52), but positive findings in other studies (52–55). The most recent meta-analysis including 45 studies did not find a significant association in the whole sample possibly due to large clinical and sociodemographic differences between the individual studies, however, a positive association was reported between the s allele and increased risk of violent suicidal behavior among substance abusers (56). Our present findings which failed to identify an effect for *5-HTTLPR* on previous suicide attempts but show an association with current suicidal ideation reflects the contradictory nature of findings concerning the involvement of this variant in suicidal behavior. Our results specifically point to the possible heterogeneity underpinning different manifestations of suicidal behavior, especially considering that in case of predictors of current suicidal risk, only suicidal ideation (Thoughts of ending your life) but not hopelessness or thoughts about death or dying showed association with *5-HTTLPR*. These latter findings also indicate that distinct genetic factors and divergent pathways may be involved in subtle differences between multiple processes contributing to the evolving risk of suicide, and draw attention to the importance of carefully differentiating between risk factors and risk phenotypes in studies concerning the genetic underpinnings of suicidal behavior.

### *5-HTTLPR* Does Not Interact With Childhood Adversities in Influencing Lifetime Suicide Attempts, Current Suicidal Ideation, Thoughts About Death, or Hopelessness

In the initial report concerning the role of the *5-HTTLPR* s allele in increasing sensitivity towards recent stressors and thus increasing the risk of depression, as well as suicidal ideation and attempts, in the face of stress exposure (22), a significant interaction between early childhood trauma during the first 10 years and *5-HTTLPR* genotype in predicting depression (but not suicide) has also been shown. Later it has been hypothesized that *5-HTTLPR* mainly mediates effects of early childhood stressors impacting neurodevelopment, leading to altered brain functioning in regions involved in mood and emotion regulation and consequentially maladaptive cognitive and behavioral patterns contributing to the manifestation of depression and risk of suicide (57, 58). While a few studies specifically looked at the interactions between early childhood stressors and *5-HTTLPR* on risk of suicidal behaviors, results have been conflicting. A significant effect of *5-HTTLPR* was found in suicide attempters reporting childhood physical and sexual but not emotional abuse in depressed patients (59), while in another study in substance abusers, association of suicide attempts with an interaction between *5-HTTLPR* genotype and early childhood trauma was reported (58). Interaction between *5-HTTLPR* and early trauma on suicidal behavior has also been found in another study in major depressive patients, however, in this study the ll genotype was found to increase risk (60). Furthermore, specifically an association with suicidal ideation and interaction between *5-HTTLPR* and early maltreatment was reported in children (61). Our results contradict these findings in reporting no interaction effect between *5-HTTLPR* and childhood adversities on either lifetime suicide attempts or current suicidal ideation, hopelessness or thoughts of death, which may, at least in part, be due to different study samples.

### *5-HTTLPR* s Allele Interacts With Recent Relationship Problems but Not Other Types of Life Events on Suicidal Ideation and Thoughts of Death

The initial results on the role of the *5-HTTLPR* short allele in increasing sensitivity towards recent stressors and its association with increased depression prevalence in the face of stress exposure (22) were followed by a large number of contradictory individual studies and meta-analyses, and the latest and largest such meta-analysis reported no significant effects (62). However, fewer studies aimed at replicating the results of Caspi and colleagues in the same study (22) reporting increased suicidal ideation and attempts in *5-HTTLPR* s allele carriers exposed to more severe recent stress. An interaction between recent stressors and *5-HTTLPR* was demonstrated in depressed patients on suicide attempts (63) with some other studies reporting negative results for suicidal ideation (64). A recent longitudinal study suggested a sex-dependent moderating effect of *5-HTTLPR* genotype of stressful life events in suicidal ideation with a strong but nonsignificant trend in female s carriers in *post hoc* tests (65). In another longitudinal study in adolescents a significant interaction between family support and *5-HTTLPR* genotype on suicidal behavior was found in boys together with a marginally significant effect in girls predicting a higher risk of suicidal attempts in s carriers with poorer social support (66). One important aspect of this study was that *5-HTTLPR* s allele increased sensitivity not only to negative environmental effects but also towards high quality positive environmental conditions, in line with the differential susceptibility theory (67). A similar study but in an elderly population reported a significant interaction between both stressful life events and deficits of social support and *5-HTTLPR* genotype on baseline prevalence and 2-year incidence of suicidal ideation (68). Finally, in a community sample of young people a significant association between non-suicidal self-injury and the interaction between s allele and interpersonal stress was reported (69).

In contrast to some of the above studies, we found no interaction between recent stress in general and *5-HTTLPR* on either suicidal ideation (thoughts of ending life), or other suicide risk factors such as hopelessness or thoughts of death. A significant interaction effect, however, emerged, in case of one specific type of recent life event. Namely, we saw a significant interaction effect with recent relationship difficulties in case of suicidal ideation and thought of death, where presence of the s allele was associated with more severe suicidal ideation when exposed to an increasing number of relationship problems.

Thus, our results implicate the involvement of *5-HTTLPR* in increasing sensitivity towards certain types recent stressful life events and impacting suicidal ideation and risk. In this sense, suicidal ideation could be regarded as a measure of the subjective impact of the difficulties which is modulated by serotonin. More importantly, our findings also support our previous hypothesis that the effect of distinct types of stressors are mediated *via* different neurobiological pathways and may contribute to the appearance of different phenotypes or clinical phenomena (44, 45, 70, 71). Previously, we reported a significant interaction with recent financial stress but not other types of life events on depressive symptoms in case of the *5-HTTLPR* (44), while in a younger subsample the s allele showed an opposite effect and was protective in case of recent social network stressors (70). In a similar model, but in case of variants in the *CNR1* gene encoding the endocannabinoid 1 receptor and the *GABRA6* gene encoding the alpha 6 subunit of the GABA-A receptor we found that not only the certain genes mediate only certain types of life events which is different in case of different genes, but also that in case of the same genetic variant, the affected outcome phenotype (in our study depression vs anxiety) may also be different in case of different types of recent stress (45). Namely, we found that *CNR1* rs7766029 in interaction with recent financial difficulties (RLE-financial) increased both depression and anxiety scores, however, *GABRA6* rs3219151 interacted with social network stressors (RLE-social) on anxiety scores and with recent personal problems (RLE-personal) on depression scores (45). Here, we extend our previous results on the *5-HTTLPR* s allele specifically mediating the effect of financial hardships on depression, with our novel findings that when exposed to recent relationship difficulties presence of the s allele leads to increased suicidal ideation and thoughts about death.

Altogether, our present results can be interpreted as a contribution to previous studies postulating a role for 5-HTTLR in suicidal behavior and risk, specifying that the existing effect may be obscured by the fact that *5-HTTLPR* has an impact only on certain phenotypes of suicidal behavior and mediates the effects of only certain types of stressors.

### *5-HTTLPR*: No Effect on Hopelessness, an Independent Factor of Suicide Risk

Finally, we must mention that *5-HTTLPR* showed no effect either directly or in interaction with either childhood or current stressors on hopelessness, which has long been established as an independent cognitive risk factor and predictor for suicide (72, 73) and specifically for suicidal ideation (74). This lack of association even as a main effect on the one hand contradicts a previous report of our group in an independent sample reporting an association between *5-HTTLPR* and hopelessness as measured by the Beck Hopelessness Scale (24), and a subsequent study where hopelessness was also associated with *5-HTTLPR* in men with cardiovascular disease (75), while on the other hand our present findings suggest that *5-HTTLPR* is involved in suicidal behavior and specifically ideation not *via* hopelessness but other processes or trait or state markers, such as for example increased aggression.

### Limitations

There are several limitations of our study which must be mentioned. First, our assessment concerning recent life events and current suicidal ideation was cross-sectional, thus we could not evaluate the longitudinal effects of recent life events on suicidal behavior. Therefore, it is possible that suicidal ideation or behavior occurring in a longer time following very recent life events could not be identified in our study. Also, we did not determine the timing of recent life events relative to current suicidal ideation. Second, RLE and CHA were recorded retrospectively and are thus subject to recall bias. Third, we only subcategorized recent life events into four, although validated, categories, it is therefore possible that some categories should be further refined. Although correlations between different categories of life events reported in our previous studies were weak, it is also possible that in isolated cases these life events are not independent of each other, which may influence the results. Fourth, all our measures, including current suicide risk markers, previous suicide attempts as well as childhood adversities and current life events are based on self-report. Fifth, our study sample is a general, non-epidemiological and non-representative population sample based on volunteers, therefore may be subject to sampling bias. Sixth, we used two geographically different subsamples in our study, and ancestry was not assessed in the present study using molecular methods such as whole-genome SNP genotyping. Although to consider this, we used population as a covariate in all our statistical analyses, there may exist subtle genetic differences both between the two subsamples and also within each sample due to population stratification which may lead to spurious effects. Finally, we would like to emphasize the exploratory nature of our analyses and urge replication of the results presented here in other cohorts.

## Conclusion

In summary, our present study supports the involvement of *5-HTTLPR* in suicidal behavior following previous conflicting results. Our findings show that *5-HTTLPR* is involved in only certain aspects of the suicide spectrum, namely suicidal ideation but not previous suicide attempts, and that it interacts with only with certain types of recent stress but not childhood adversities. This suggests that existing effects may be obscured in several studies not differentiating carefully between suicidal phenotypes and stressor types. Furthermore, our results also support our previous findings that specific genes and variants, such as the *5-HTTLPR* may only mediate the effect of certain types of stressors and may lead to the emergence of different phenotypes depending on the type of stressor. Thus, our results emphasize the importance of differentiating between types of stress in gene-environment studies to avoid obscuring existing associations, and also suggest that future sophisticated models of predicting and preventing suicide should include highly specific gene-environment interaction pathways. Further studies should focus on the prospective association between distinct types of current stressors and suicide attempts and completed suicides in relation to *5-HTTLPR* genotype, and should also attempt to investigate whether the current findings can be employed in preventive approaches to decrease suicidal ideation in short allele carriers after exposed to distinct types of stress.

## Data Availability Statement

Raw data comprising the basis of the presented analyses and supporting the conclusions of this article are available at https://doi.org/10.6084/m9.figshare.12214748.v1.

## Ethics Statement

The studies involving human participants were reviewed and approved by the Scientific and Research Ethical Review Board of the Medical Research Council. The participants provided their written informed consent to participate in this study.

## Author Contributions

XG, GB, and GJ designed and conceptualized the study and collected the data. SK, DT, NE, ZG, and SS and participated in statistical analyses. All authors participated in interpreting the data. XG, JB, and DT wrote the first draft of the manuscript. DT and SS created figures. All authors participated in developing further and final versions of manuscript. All authors contributed to the article and approved the submitted version.

## Funding

This study was supported by the Sixth Framework Program of the European Union (NewMood, LSHM-CT-2004-503474); the Hungarian Academy of Sciences (MTA-SE Neuropsychopharmacology and Neurochemistry Research Group); the Hungarian Brain Research Program (Grants: 2017-1.2.1-NKP-2017-00002; KTIA_13_NAPA-II/14); the National Development Agency (Grant: KTIA_NAP_13-1-2013- 0001); the Hungarian Academy of Sciences, Hungarian National Development Agency, Semmelweis University, and the Hungarian Brain Research Program (Grant: KTIA_NAP_13-2- 2015-0001) (MTA-SE-NAP B Genetic Brain Imaging Migraine Research Group); the ITM/NKFIH Thematic Excellence Programme, Semmelweis University; and the SE-Neurology FIKP grant of EMMI. Xenia Gonda is supported by the Janos Bolyai Research Fellowship of the Hungarian Academy of Sciences. Xenia Gonda is supported by ÚNKP-19-4-SE-19 and Sara Sutori by ÚNKP-19-1-1-PPKE-63 New National Excellence Program of the Ministry for Innovation and Technology. The sponsors had no further role in the study design; in the collection, analysis and interpretation of data; in the writing of the report; and in the decision to submit the paper for publication.


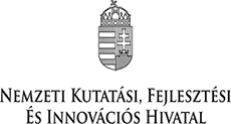
    
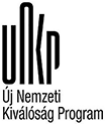


## Conflict of Interest

BD has recently received fees for scientific advice from Autifony and he has share options in P1vital. IA has received consultancy fees from Servier, Alkermes, Lundbeck/Otsuka, and Janssen, an honorarium for speaking from Lundbeck and grant support from Servier and AstraZeneca.

The remaining authors declare that the research was conducted in the absence of any commercial or financial relationships that could be construed as a potential conflict of interest.
